# Kyphectomy followed by self-sliding pedicle screw and translumbosacral rod impaction and fixation: a novel growth-friendly technique in myelomeningocele patients

**DOI:** 10.1007/s43390-024-01036-1

**Published:** 2025-01-11

**Authors:** Alderico Girão Campos Barros, Diogo R. Noronha, Luis E. Carelli, David L. Skaggs

**Affiliations:** 1 Department of Spine Surgery, National Institute of Orthopedics and Traumatology, INTO, Avenida Brasil 500 Caju, Rio de Janeiro, RJ 20940-070 Brazil; 2 Department of Spine Surgery, National Institute of Orthopedics and Traumatology, INTO, Rio de Janeiro, Brazil; 3 Pediatrics and Neurosurgery, Cedars Sinai Medical Center, Los Angeles, CA USA

**Keywords:** Kyphectomy, Growth-friendly, Myelomenigocele

## Abstract

**Introduction:**

Congenital lumbar kyphosis is present in about 15% of patients with myelomeningocele. Worsening of deformity with complications such as chronic skin ulcers and bone exposure is common. In patients under 8 years of age, treatment becomes even more challenging: in addition to resecting the apex of the kyphotic deformity, we should ideally stabilize the spine with fixation methods that do not interrupt the growth of the rib cage, associated with the challenging pelvic fixation in this population. The emergence of growth-friendly techniques has greatly benefited patients with early-onset deformity, allowing for correction and control of deviation without interrupting trunk growth, which is often already compromised in these patients. We describe the surgical technique and present preliminary clinical outcomes for a novel approach which combines self-sliding screws that allow for trunk growth with impaction of translumbosacral rods for distal fixation.

**Methodology:**

Candidates for the technique were patients with myelomeningocele and congenital lumbar kyphosis, inability to assume supine position, and a history of skin ulcers, recurrent osteomyelitis and poor weight gain. They all lacked sensory or motor function below the level of the myelomeningocele. They underwent the same reconstruction technique after kyphectomy which combines self-sliding screws that allow for trunk growth with impaction of the translumbosacral rods for distal fixation.

**Results:**

**Case 1:** Female; 4.5 years old at surgery; 5 year follow-up. 1 complication: loosening of one blocker. The child is doing well and did not require surgical revision. Mean growth per year: 9.5 mm. **Case 2:** Male; 7.4 Years old at surgery; 4 year follow-up. 1 complication: post-surgical infection which required 2 debridements in the operating room and prolonged antibiotic therapy. Mean growth per year: 6 mm. **Case 3:** Female; 5.5 Years old at surgery; 27 month follow-up. No complications reported so far. Mean growth per year: 9.42 mm. None of the cases showed signs of sacral osteolysis or rod migration.

**Discussion / Conclusion:**

To our knowledge, this is the first study that combines sliding screws with translumbosacral rod impaction. Although this technique has proven to be safe and effective, we are aware that the number of cases is limited and the follow-up is short. Further studies are necessary to confirm the method.

## Introduction

Myelomeningocele is a multisystem disease, which makes its treatment quite complex. Among the associated conditions, congenital lumbar kyphosis, present in about 15% of patients, is one of the most difficult deformities to manage. It usually already has a high angular value from birth, and in addition to being rigid, tends to progress rapidly [1, 2]. When assuming the seated position, there is an increase in the magnitude of the curve and complications such as chronic skin ulcers, bone exposure, osteomyelitis, pain from iliocostal impact, respiratory compromise, postural and hygienic difficulties, as well as self-image impairment often arise [2–5]. The indication and execution of surgery should be very careful, as the treatment is challenging, and the complication rate is high [6–9].

Patients commonly present with weight and stature deficits, central nervous system alterations, poor skin condition, distorted anatomy and poor bone quality. Once indicated, surgical intervention should be early, allowing for greater correction with less morbidity. [10] On the other hand, in patients under 8 years of age, treatment becomes even more challenging: in addition to resecting the apex of the kyphotic deformity, we should ideally stabilize the spine with fixation methods that do not interrupt the growth of the rib cage, allowing for proper lung development and improved life expectancy, while addressing the difficulties of pelvic fixation in these patients [6, 11, 12].

Since Sharrard’s initial publication in 1968 [13], describing the treatment of neonates with congenital lumbar kyphosis in myelomeningocele, there has been considerable progress in understanding the natural history of the disease as well as advances in the implants used in pediatric spinal deformity surgery. However, this evolution relied on different techniques, most of which involve vertebral fusion, which while improving patient outcomes, still have consequences that need to be addressed [14–16].

The emergence of growth-friendly techniques has greatly benefited patients with early-onset deformity, allowing for correction and control of deviation without interrupting trunk growth, which is often already compromised in these patients [12, 17–19]. We also know that while these methods bring benefits, they have a considerable complication rate and that the presence of kyphosis is a complicating factor for surgical correction [20, 21].

In this case series report, we describe the surgical technique and present preliminary clinical outcomes for a novel combination of techniques in which self-sliding screws are used to allow for trunk growth, impaction of the translumbosacral rods reduces tissue dissection, and kyphectomy corrects the deformity.

## Clinical cases

All patients had a diagnosis of myelomeningocele with congenital lumbar kyphosis, inability to assume supine position and a history of recurrent osteomyelitis and poor weight gain. All three patients had no sensory or motor function below the level of the myelomeningocele. The legal guardians of the patients consented to participate in the study.

### Surgical technique

**Fig. 1:**
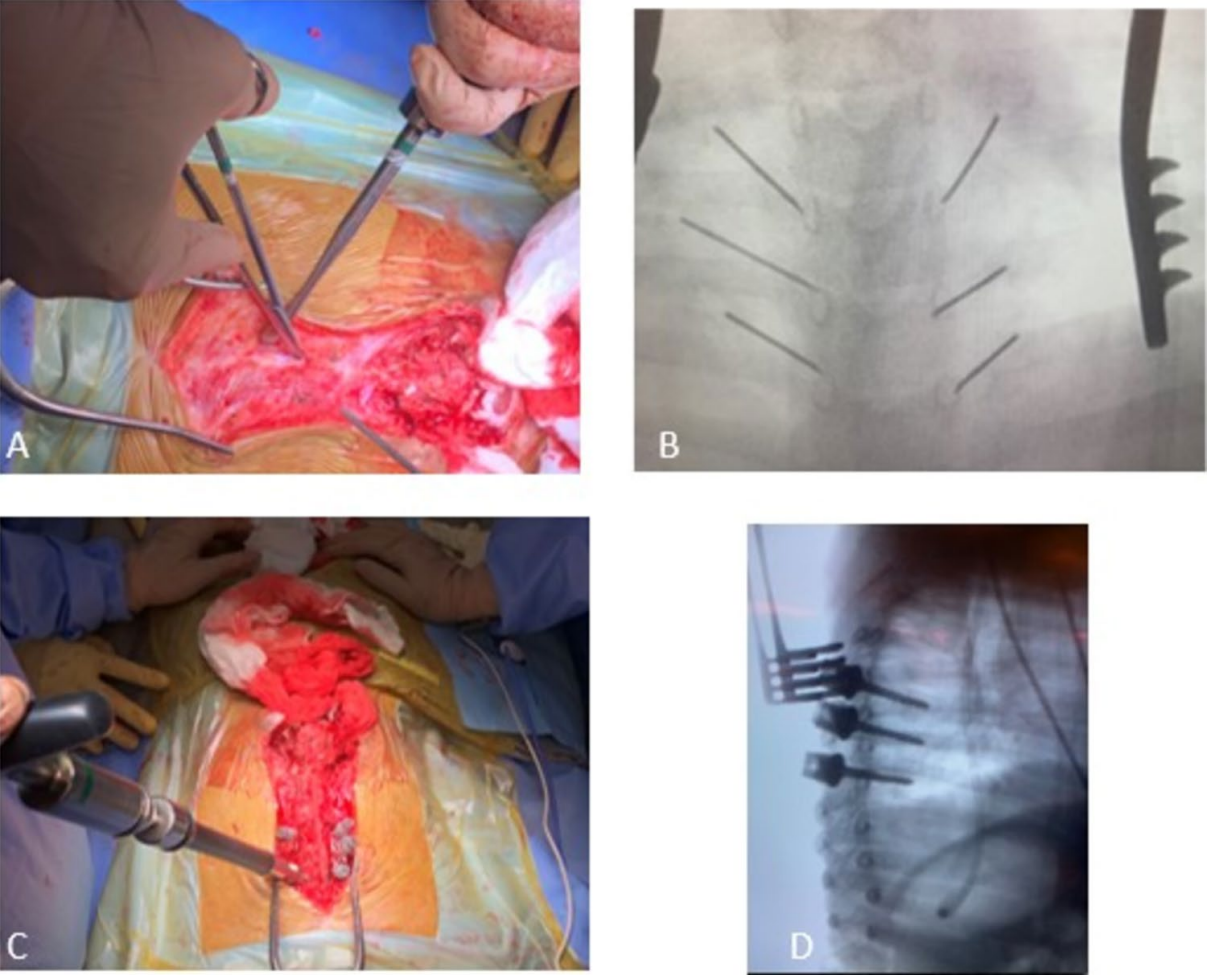
**A** Dissection of the thoracic segment was limited to the fascia without exposing the periosteum; **B** Pedicles were marked using fluoroscopy, **C** and the proximal screws were passed intermuscularly. **D** Fluoroscopic imaging in lateral was used to confirm the appropriate length of the pedicle screws

**Proximal portion:** The patient is received in a “latex-free” surgical environment, and the surgery is performed under general anesthesia with neurophysiological monitoring. After positioning the patient in the prone position and applying sterile drapes, a median longitudinal incision is made in the proximal thoracic spine up to the level of the congenital defect. The muscular fascia is visualized but not opened to avoid exposure of the periosteum, inadvertent bone fusion, and consequent compromise to trunk growth. With the aid of fluoroscopy, the pedicles of three to four vertebral levels in the middle thoracic spine are identified, and transmuscular placement of bilateral 4.5-mm diameter pedicle screws (the standard diameter used to receive adult rods) is performed, reaching the maximum length possible. (Fig. 1) It is important to insert the screws as deep as possible into the muscles, to avoid problems with prominence in the skin.

**Distal portion:** Exposure of the congenital kyphotic defect is performed with a smaller dissection when compared to traditional methods, in our technique there is no need to expose the caudal lumbar vertebrae, sacrum and iliac. The distal portion of the apex of the kyphosis is dissected subperiosteally. The dural sac is identified and meticulously dissected to minimize the possibility of dural injury and cerebrospinal fluid leakage.

**Fig. 2:**
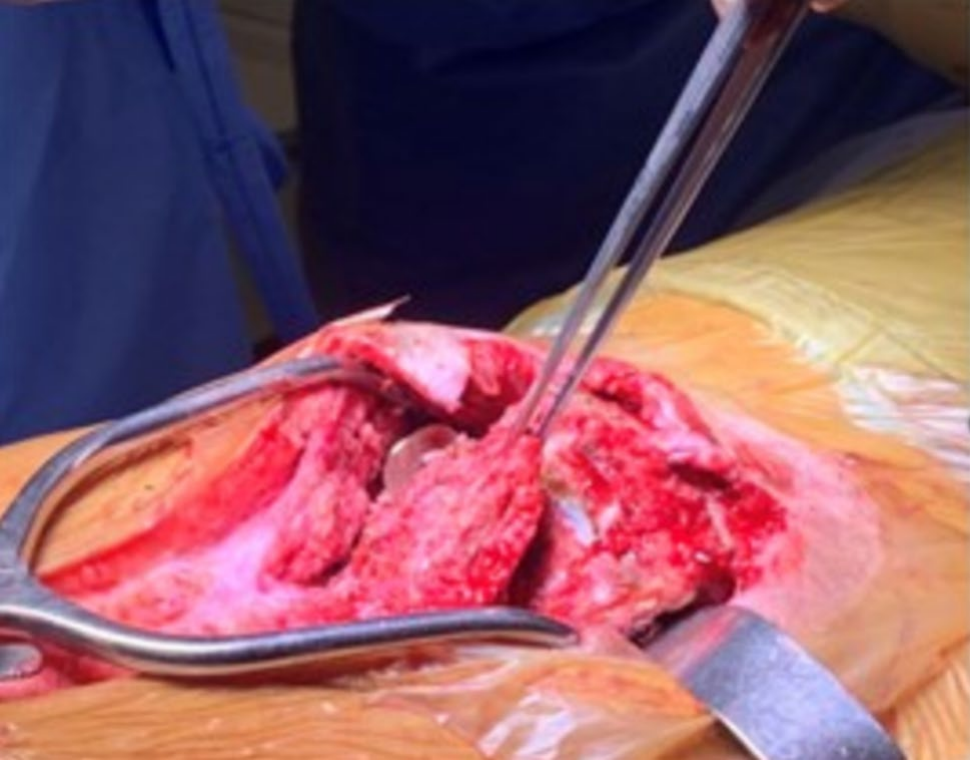
Identification and retraction of the dural sac

In none of the cases was it necessary to ligate the dural sac, which was only retracted (Fig. 2). Dissection of the lateral and anterior portions of the apical vertebral bodies is performed with careful bone removal and segmental arteries are ligated. In cases of hyperkyphosis, large vessels do not usually accompany the bone deformity [22], therefore there is a good anterior safety margin to resection after the isolation and protection of these structures.

The number of vertebrae to be resected is decided “à la carte” during surgery and varies from two to three vertebral levels, always those located cephalad to the apex of the kyphotic defect, to preserve adequate bone stock for distal spinal stabilization. During vertebrectomies, it is important to avoid excessive removal of bone tissue. The extent of the bone resection should be executed vertebra by vertebra to avoid a gap and assure bone to bone contact after deformity correction. To attain this approximation, manual control of the resection ends is performed using a cantilever maneuver.

**Fig. 3:**
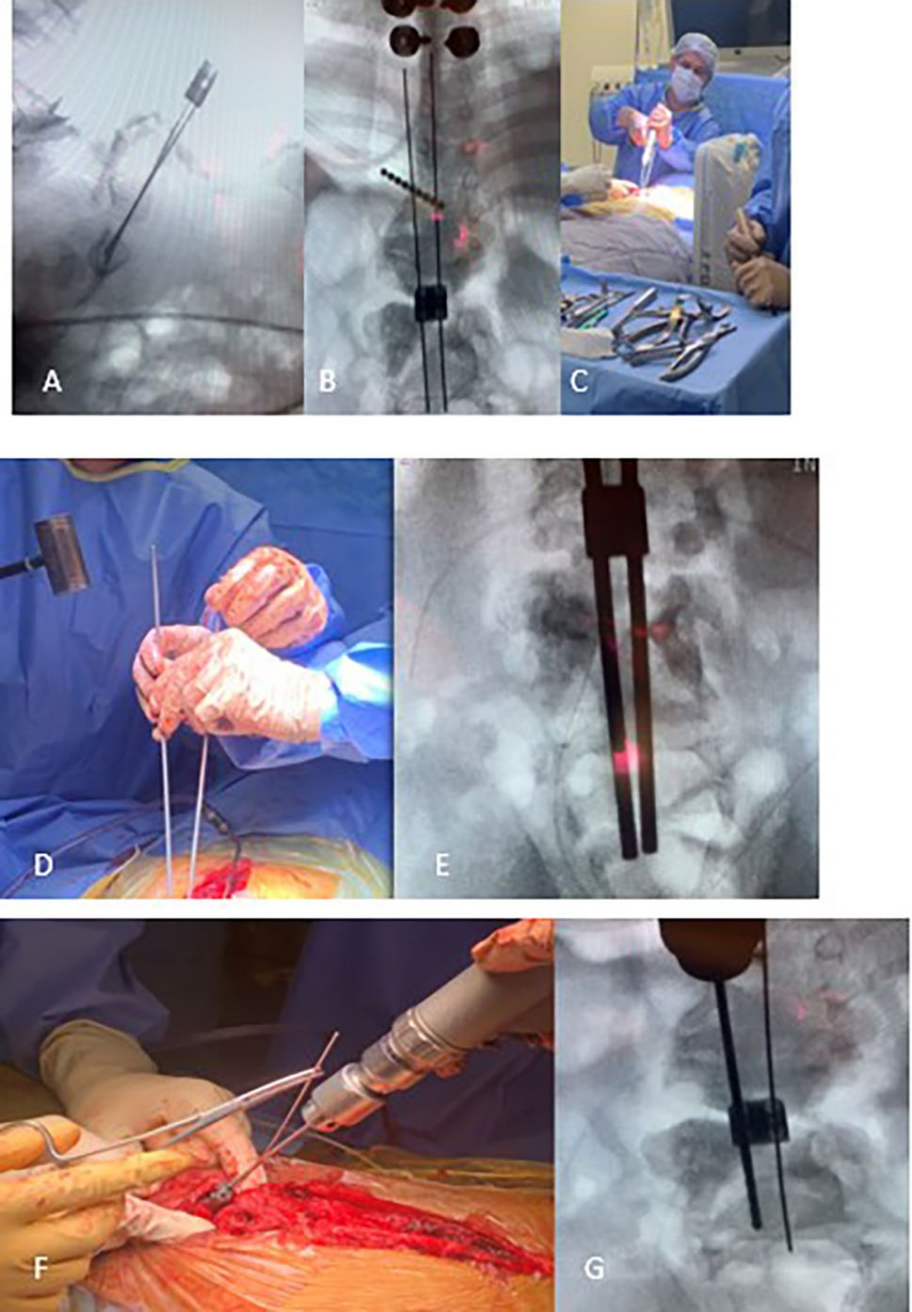
**A**–**C** Intraoperative fluoroscopic image of guide wires being passed through the dowel to block possible rod migration; **D**–**E** Insertion of cannulated drill bit into vertebral bodies of the distal pole of the construct G **F**–**G** Impaction of the rods using a hammer

After the vertebrectomies, a 1.5-mm diameter guide wire is passed through a domino in the center of the upper endplate of the most proximal lumbar vertebral body belonging to the distal stump under fluoroscopic control in both AP and lateral views. The trajectory of the guide wire should traverse as many lumbar vertebrae as possible and pass through the anterior cortical bone of S1 or S2 by approximately 0.5 cm. Once the correct positioning of the guide wire is confirmed, a 2.8 mm cannulated drill bit is passed over the wire under fluoroscopic control in both AP and lateral views. After confirming the correct positioning of the hole drilled to receive the rod, a 4.0 mm diameter titanium rod is carefully impacted into place using a controlled hammering technique, taking care to avoid vertebral fracture, as these patients typically have poor bone quality (Fig. 3).

The rods are cut in such a way that their final lengths extend 3 to 4 proximal levels beyond the upper instrumented vertebra, allowing for guided trunk growth. Kyphosis is corrected with a cantilever maneuver, the pedicle screw blockers are placed to maintain the correction, and the dowel is locked. Finally, we perform wound closure by layers and place a drain. Such patients did not require postoperative immobilization or skin flap closure during follow-up.

**Fig. 4:**
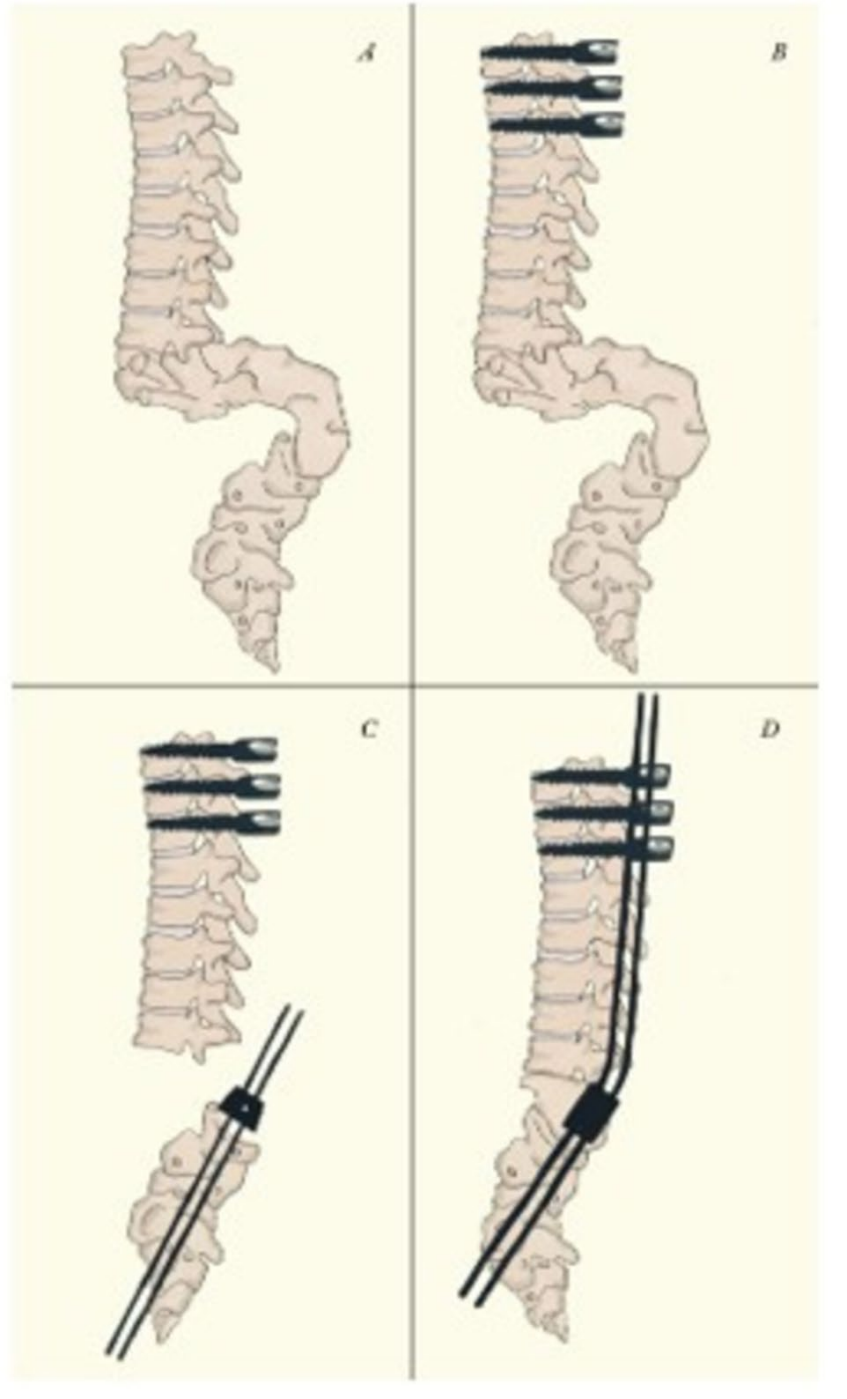
Illustration of surgical technique. **A** preoperative aspect of spinal deformity. **B** Transmuscular instrumentation of 3 to 4 bilateral 4.5 mm diameter pedicle screws in the middle thoracic spine, reaching the maximum depth possible. **C** After the vertebrectomies, a 1.5 mm diameter guide wire is passed through a domino in the center of the upper endplate of the most proximal lumbar vertebral body belonging to the distal stump. Once the correct positioning of the guide wire is confirmed, a 2.8 mm cannulated drill bit is passed. After confirming the correct positioning of the hole drilled to receive the rod, a 4.0 mm diameter titanium rod is carefully impacted into place. **D** The rods are cut in such a way that their final lengths extend 3 to 4 proximal levels beyond the upper instrumented vertebra, allowing for guided trunk growth. Kyphosis is corrected with a cantilever maneuver, the pedicle screw blockers are placed to maintain the correction, and the domino is locked. As the rods are not locked by the thoracic screw lockers, trunk growth is allowed by the rod sliding. There is no need for more distal dissection of the spine and pelvic instrumentation, which can be technically demanding in this group of patients

The surgical strategy is summarized in Fig. 4.

## Results

**Fig. 5:**
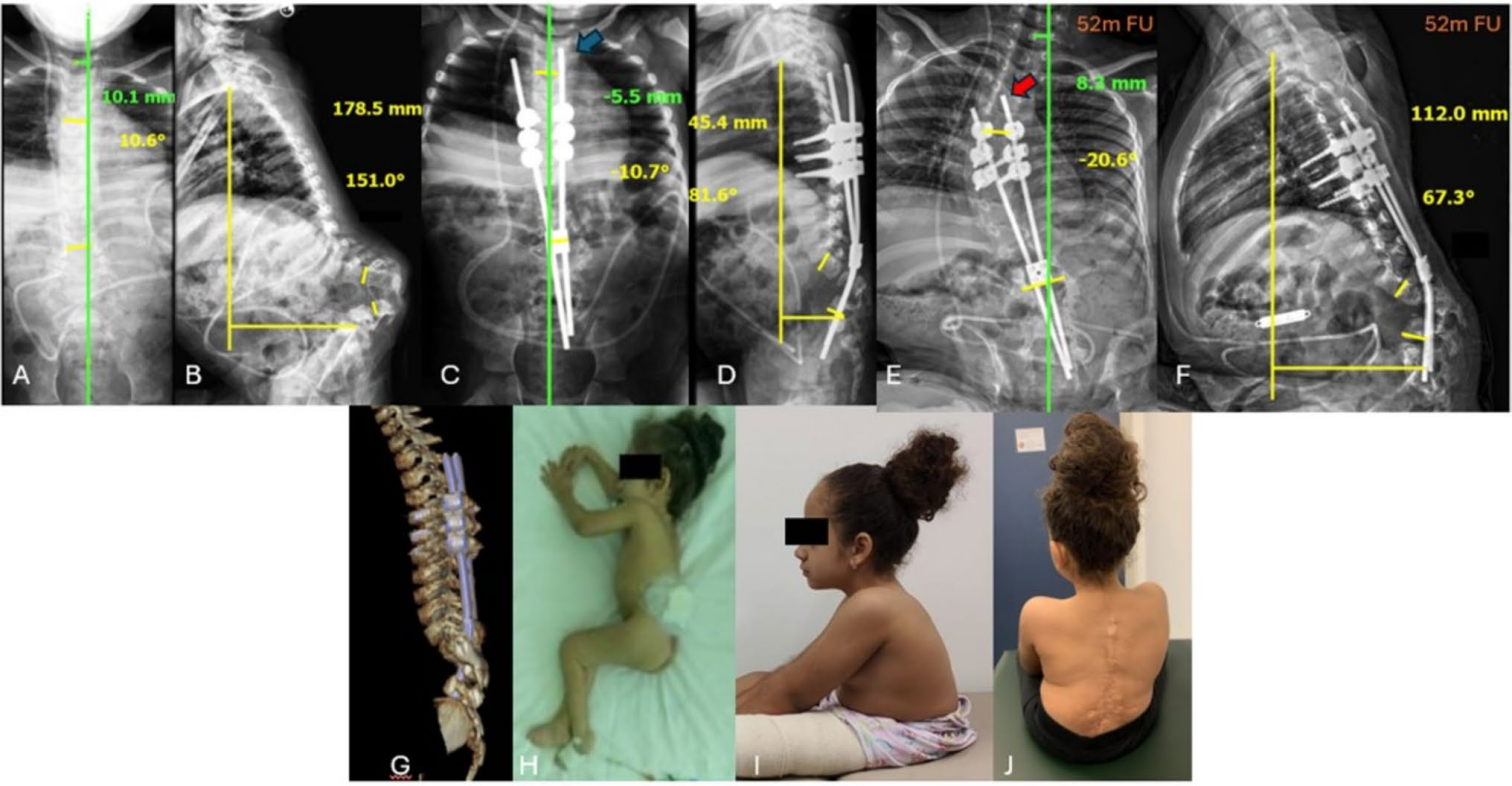
**A**, **B** AP and lateral preoperative radiographies showing serious lumbar kyphosis; **C**, **D** immediate postoperative radiographies presents the length of the rod above the proximal screws [blue arrow], allowing trunk growth; **E**, **F** Panoramic radiograph on follow-up showing a decrease in the length of the rod above the proximal screws [red arrow], what was allowed by the self-sliding technique after 42 months of follow up; **G** Postoperative 3D tomographic reconstruction; **H** Clinical feature of patient 1 before procedure; **I**, **J** Clinical feature of patient 1 at 52 months of follow-up

**Case 1:** Female; 4.5 years old at surgery; preoperative cobb angle: 10.6º; coronal alignment 10.1 mm; SVA 178.5 mm; regional kyphosis 151º; upper instrumented levels: T5, T6 and T7; 3 resected vertebrae; immediate postoperative cobb angle 10.7º; coronal alignment 5.5 mm; SVA 45.4 mm; regional kyphosis 81.6º; 5 year follow-up: Cobb angle: 20.6º; coronal alignment 8.3 mm; SVA 112 mm; regional kyphosis: 67.3º. 1 complication: loosening of one blocker. The child is doing well and did not require surgical revision (Fig. 5).

**Fig. 6:**
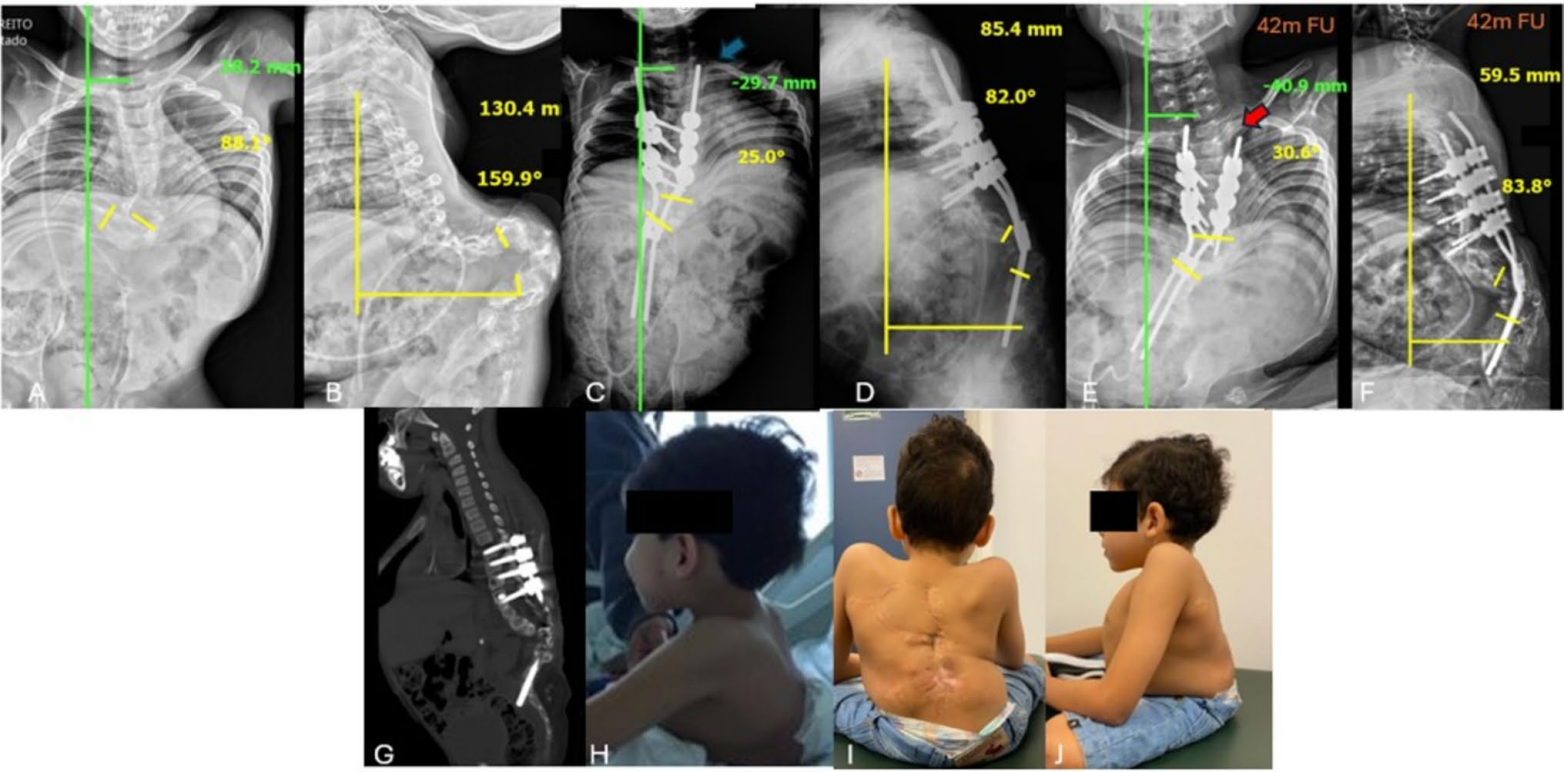
**A**, **B** AP and lateral preoperative radiographies showing serious lumbar kyphosis; **C**, **D** Immediate postoperative radiographies presents the length of the rod above the proximal screws [blue arrow], allowing trunk growth; **E**, **F** Panoramic radiograph on follow-up showing a decrease in the length of the rod above the proximal screws [red arrow), what was allowed by the self-sliding technique after 42 months of follow up; **G** Postoperative Sagittal CT; **H** Clinical feature of patient 2 before procedure; **I**, **J** Clinical feature of patient 2 at 42 months of follow-up

**Case 2:** Male; 7.4 years old at surgery; preoperative Cobb angle 88.1º; coronal alignment 38.2 mm; SVA 130.4 mm; regional kyphosis 159.9º; upper instrumented levels: T4, T5, T6 and T7; 3 resected vertebrae; immediate postoperative Cobb angle 25º; coronal alignment 29.7 mm; SVA 85.4 mm; regional kyphosis 82º; 4 year follow-up: Cobb angle 30.6º; coronal alignment 40.9 mm; SVA 59.5 mm; regional kyphosis 83.8º. 1 complication: post-surgical infection which required 2 debridements in the operating room and prolonged antibiotic therapy (Fig. 6).

**Fig. 7:**
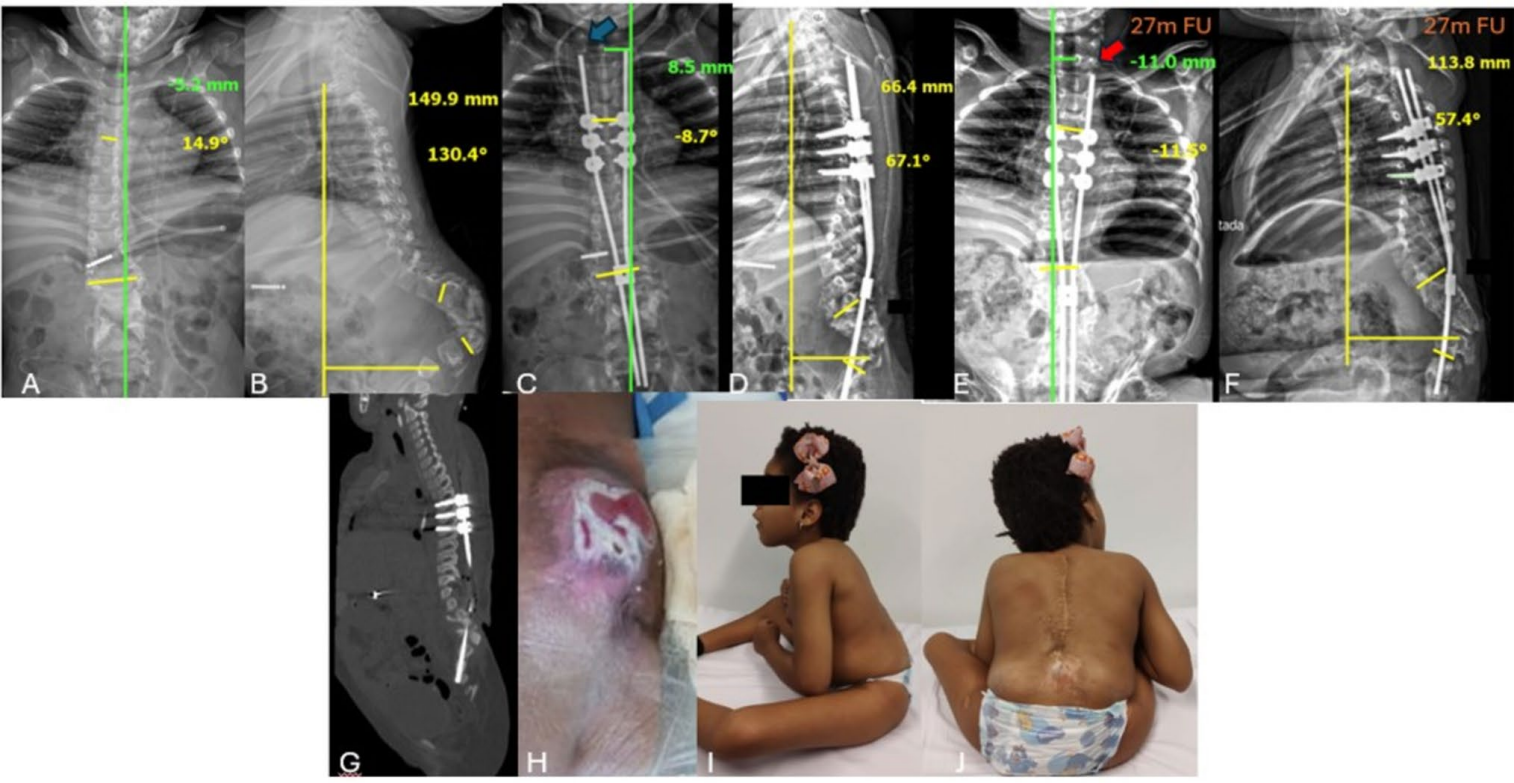
**A**, **B** AP and lateral preoperative radiographies showing serious lumbar kyphosis; **C**, **D** immediate postoperative radiographies presents the length of the rod above the proximal screws [blue arrow], allowing trunk growth; **E**, **F** Panoramic radiograph on follow-up showing a decrease in the length of the rod above the proximal screws [red arrow], what was allowed by the self-sliding technique after 27 months of follow up; **G** Postoperative Sagittal CT; **H** Clinical feature of patient 3 before procedure; **I**, **J** Clinical feature of patient 3 at 27 months of follow-up

**Case 3:** Female; 5.5 years old at surgery; preoperative cobb angle 14.9º; coronal alignment 5.2 mm; SVA 149.9 mm; regional kyphosis 130.4º; upper instrumented levels: T4, T5 and T6; 2 resected vertebrae; immediate postoperative cobb angle 8.7º; coronal alignment 8.5 mm; SVA 66.4 mm; regional kyphosis 67.1º; 27 month follow-up: cobb angle 11.5º; coronal alignment 11 mm; SVA 113.8 mm; regional kyphosis 57.4º. No complications reported so far (Fig. 7).

The radiographic measurements are summarized in Table 1.

**Table 1:** Radiographic measurements as Cobb angle, Coronal alignment, SVA and Regional Kyphosis in comparison at pre-operative, immediative post-operative and final follow up

	Cobb			Coronal Alignment			SVA			Regional Kyphosis			Follow -up
	Pre-op	Immediative Post-op	Last FU	Pre-op	Immediative Post-op	Last FU	Pre-op	Immediative Post-op	Last FU	Pre-op	Immediative Post-op	Last FU	
Patient 1	10.6	10.7	20.6	10.1mm	5.5mm	8.3mm	178.5mm	45.4mm	112mm	151	81.6	67.3	52 months
Patient 2	88.1	25	30.6	38.2mm	29.7mm	40.9mm	130.4mm	85.4mm	59.5mm	159.9	82	83.8	42 months
Patient 3	14.9	8.7	11.5	5.2mm	8.5mm	11mm	149.9mm	66.4mm	138.8mm	10.4	67.1	57.4	27 months

None of the cases showed signs of sacral osteolysis, rod migration, problems related to metal debris and prominence of screws in the skin.

## Discussion

Meningomyelocele remains a challenge for spinal surgery. One of the many spectrums of this disease is congenital lumbar kyphosis, which is usually early and severe. The indication for surgery in these patients persists due to the poor outcome of conservative treatment. In young children, proper management becomes even more challenging as it requires vertebral resection at the apex of the kyphosis followed by stabilization using a method that allows trunk growth after the procedure. Despite significant advances in this field, the existing literature is still limited: the first article comparing the results of meningomyelocele patients undergoing apical resection followed by instrumentation using a growth-friendly technique was published less than a decade ago [23].

In the present article, we present a new combination of surgical techniques as an option for managing congenital lumbar kyphosis in meningomyelocele. We believe that this approach can lead to a reduction in typical disease complications while not interrupting trunk growth, which is an important factor for good clinical outcomes [24]. Several important aspects of the technique should be emphasized. First, although distal fixation through translumbosacral impaction of rods has been described in the past, the literature on it is very scarce [25–27]. This lumbopelvic fixation technique allows a shorter extension of the skin incision. The patient is also spared extensive bone exposure, which is required for pelvic stabilization in traditional techniques. We believe that this reduces surgical time, bleeding, and tissue damage. Consequently, the possibility of related complications, such as wound dehiscence and infection, would also decrease [27]. Özcan et al., who operated on the same profile of patients, had postoperative wound complications in 19 out of 30 patients using iliac screws, including the need for revision for removal in four patients [28].

The use of pedicle screws as an alternative to sublaminar wires allows for greater stability in the construct with a lower chance of mechanical failure. The transmuscular instrumentation is done with screws with a diameter of 4.5–5.5 mm (standard sizes to receive adult rods) with 4.0-mm rods (standard pediatric size), so that the final torque should lock the blocker on the screw but permit the sliding of the rods to enable for trunk growth and proper lung development after surgery. This combination of screws, blockers, and rods should be tested before surgery because not all materials allow for the sliding of the rod after the blocker is locked on the screw. It is important to note that we use this adaptation because we do not have the Shilla system in our country [29].

During the follow-up of our patients, we observed that the self-sliding screws did allow for trunk growth guided by the rods. Using the same parameter as previous studies [24, 29], radiographic measurement of the T1-T12 height shows growth of 40.8 mm, 21 mm, and 18.3 mm, respectively (Table 2). The locking of the domino is crucial to prevent possible migration of the rods since the blockers of the proximal pedicle screws do not lock them in place.

**Table 2:** T1-T12 heighs in anteroposterior radiographs

	Case 1	Case 2	Case 3
Immediative postoperative (T1-T12)	145.2 mm	123 mm	154 mm
Last follow-up (T1-T12)	186 mm	144 mm	172.5 mm
Follow-up	52 months	42 months	27 months
Growth per year	9.5 mm	6 mm	9.42 mm

Bas et al. compared the techniques of growing rods and Luque-Trolley after performing kyphectomy. Although patients treated with growing rods showed a slightly higher chest growth rate, the number of complications requiring unplanned surgeries was much more significant [23]. Reoperations, an integral part of treatment with growing rods, were also a significant point in Alshaalan et al.’s study [25]. As it is a population with fragile clinical status, the morbidity of any surgical procedures as well as the anesthesia itself cannot be ignored, and the psychologic trauma for the patient and family cannot be overlooked [30]. The objective should be to reduce repeated surgical interventions, which in addition to being beneficial for the patient, is also an imperative in our setting due to the limited economic resources.

To our knowledge, this is the first study that combines sliding screws with translumbosacral rod impaction. Although this technique has proven to be safe and effective, we are aware that the number of cases is limited, and the follow-up is short. Further studies are necessary to confirm the method.

## Declarations

### Informed concent

Written informed consent was obtained from the parents.

## References

[1] Hoppenfeld S (1967) Congenital kyphosis in myelomeningocele. J Bone Joint Surg Br 49:276–280 5338247

[2] Carstens C, Koch H, Brocai DR, Niethard FU (1996) Development of pathological lumbar kyphosis in myelomeningocele. J Bone Joint Surg Br 78(6):945–950 8951012 10.1302/0301-620x78b6.1272

[3] Sharrard WJ (1971) Lumbar and sacral ulceration in older paraplegic children with special reference to progressive kyphosis. Proc R Soc Med 64(11):1145–1147 4943378 10.1177/003591577106401114 PMC1812071

[4] Garg S, Oetgen M, Rathjen K, Richards BS (2011) Kyphectomy improves sitting and skin problems in patients with myelomeningocele. Clin OrthopRelat Res 469(5):1279–1285. 10.1007/s11999-010-1650-8 10.1007/s11999-010-1650-8 PMC3069289 21042894

[5] Dunn RN, Bomela LN (2016) Kyphectomy in children with severe myelomeningocele-related kyphosis. SpineDeform 4(3):230–236. 10.1016/j.jspd.2015.11.006 10.1016/j.jspd.2015.11.006 27927508

[6] McMaster MJ (1988) The long-term results of kyphectomy and spinal stabilization in children with myelomeningocele. Spine 13(4):417–424 3406851 10.1097/00007632-198804000-00009

[7] Ko AL, Song K, Ellenbogen RG, Avellino AM (2007) Retrospective review of multilevel spinal fusion combined with spinal cord transection for treatment of kyphoscoliosis in pediatric myelomeningocele patients. Spine 32(22):2493–2501 18090091 10.1097/BRS.0b013e3181573b11

[8] Heydemann JS, Gillespie R (1987) Management of myelomeningocele kyphosis in the older child by kyphectomy and segmental spinal instrumentation. Spine 12(1):37–41 3576353 10.1097/00007632-198701000-00007

[9] Niall DM, Dowling FE, Fogarty EE, Moore DP, Goldberg C (2004) Kyphectomy in children with myelomeningocele: a long-term outcome study. J PediatrOrthop 24(1):37–44 10.1097/00004694-200401000-00008 14676532

[10] Luhmann SJ, Furdock R (2019) Preoperative variables associated with respiratory complications after pediatric neuromuscular spine deformity surgery. SpineDeform 7(1):107–111 10.1016/j.jspd.2018.05.005 30587301

[11] Campbell RM Jr, Smith MD (2007) Thoracic insufficiency syndrome and exotic scoliosis. J Bone Joint Surg Am 89(Suppl 1):108–122. 10.2106/JBJS.F.00270 17272428 10.2106/JBJS.F.00270

[12] Skaggs DL, Akbarnia BA, Flynn JM, Myung KS, Sponseller PD, Vitale MG, Chest Wall and Spine Deformity Study Group; Growing Spine Study Group; Pediatric Orthopaedic Society of North America; Scoliosis Research Society Growing Spine Study Committee (2014) A classification of growth friendly spine implants. J PediatrOrthop. 34(3):260–274 10.1097/BPO.0000000000000073 23995146

[13] Sharrard WJ (1968) Spinal osteotomy for congenital kyphosis in myelomeningocele. J Bone Joint Surg Br 50(3):466–471 (**PMID: 4882211**) 4882211

[14] Petersen PA, Marcon RM, Letaif OB, Mello Santos MA, Oliveira RG, de Barros P, Filho TE, Cristante AF (2020) Does kyphectomy improve the quality of life of patients with myelomeningocele? Clin OrthopRelat Res 478(1):104–111 10.1097/CORR.0000000000000976 PMC7000045 31567706

[15] El-Hawary R (2020) CORR insights®: does kyphectomy improve the quality of life of patients with myelomeningocele? Clin OrthopRelat Res 478(1):112–113. 10.1097/CORR.0000000000001058 10.1097/CORR.0000000000001058 PMC7000057 31764316

[16] Kabirian N, Akbarnia BA, Pawelek JB, Alam M, Mundis GM Jr, Acacio R, Thompson GH, Marks DS, Gardner A, Sponseller PD, Skaggs DL, Growing Spine Study Group (2014) Deep surgical site infection following 2344 growing-rod procedures for early-onset scoliosis: risk factors and clinical consequences. J Bone Joint Surg Am 96(15):e128 25100781 10.2106/JBJS.M.00618

[17] Moe JH, Kharrat K, Winter RB, Cummine JL (1984) Harrington instrumentation without fusion plus external orthotic support for the treatment of difficult curvature problems in young children. Clin OrthopRelat Res 185:35–45 6705397

[18] Luque ER (1982) Paralytic scoliosis in growing children. Clin OrthopRelat Res 163:202–209 7067255

[19] Campbell RM Jr (2013) VEPTR: past experience and the future of VEPTR principles. Eur Spine J. 10.1007/s00586-013-2671-2 23354777 10.1007/s00586-013-2671-2 PMC3616470

[20] Rinsky LA, Gamble JG, Bleck EE (1985) Segmental instrumentation without fusion in children with progressive scoliosis. J PediatrOrthop 5(6):687–690 10.1097/01241398-198511000-00011 4066943

[21] Akbarnia BA, Marks DS, Boachie-Adjei O, Thompson AG, Asher MA (2005) Dual growing rod technique for the treatment of progressive early-onset scoliosis: a multicenter study. Spine 30(17):S46-57 16138066 10.1097/01.brs.0000175190.08134.73

[22] Fromm B, Carstens C, Niethard FU, Lang R (1992) Aortography in children with myelomeningocele and lumbar kyphosis. J Bone Joint Surg Br 74(5):691–694 1527114 10.1302/0301-620X.74B5.1527114

[23] Bas CE, Preminger J, Olgun ZD, Demirkiran G, Sponseller P, Yazici M, Growing Spine Study Group (2015) Safety and efficacy of apical resection following growth-friendly instrumentation in myelomeningocele patients with gibbus: growing rod versus luque trolley. J PediatrOrthop 35(8):98–103 10.1097/BPO.0000000000000419 25705808

[24] Karol LA (2011) Early definitive spinal fusion in young children: what we have learned. Clin OrthopRelat Res 469(5):1323–1329 10.1007/s11999-010-1622-z PMC3069259 20957466

[25] Alshaalan KS, Howard JJ, Alshangiti AK, Alkhalife YI, Aleissa S, Al Sayegh SO (2019) Kyphectomy in myelomeningocele for severe early-onset kyphosis using distal intravertebral fixation and thoracic growing rods. J Am AcadOrthop Surg Glob Res Rev 3(9):e006 10.5435/JAAOSGlobal-D-19-00006 PMC6860136 31773078

[26] Torode I, Godette G (1995) Surgical correction of congenital kyphosis in myelomeningocele. J PediatrOrthop 15(2):202–205 7745094

[27] Comstock SA, Cook PC, Leahey JL, El-Hawary R, Hyndman JC (2011) Posterior kyphectomy for myelomeningocele with anterior placement of fixation: a retrospective review. Clin OrthopRelat Res 469(5):1265–1271. 10.1007/s11999-010-1611-2 10.1007/s11999-010-1611-2 PMC3069298 20949380

[28] Özcan Ç, Polat Ö, Alataş İ, Çamur S, Sağlam N, Uçar BY (2020) Clinical and radiological results of kyphectomy and sliding growing rod surgery technique performed in children with myelomeningocele. J OrthopSurg Res 15(1):576 10.1186/s13018-020-02099-2 PMC7708111 33261632

[29] McCarthy RE, Luhmann S, Lenke L, McCullough FL (2014) The Shilla growth guidance technique for early-onset spinal deformities at 2-year follow-up: a preliminary report. J PediatrOrthop 34(1):1–7 10.1097/BPO.0b013e31829f92dc 23934092

[30] Suliman S, Mkabile SG, Fincham DS, Ahmed R, Stein DJ, Seedat S (2009) Cumulative effect of multiple trauma on symptoms of posttraumatic stress disorder, anxiety, and depression in adolescents. ComprPsychiatry 50(2):121–127 10.1016/j.comppsych.2008.06.006 19216888

